# Derby and Oxford

**Published:** 1891-06-06

**Authors:** 


### The Doctor's Armchair.

DERBY AND OXFORD.

When the Queen laid the foundation stone of the new Town and County Hospital, at Derby, the other day, she said: "In laying the foundation stone of the new building which you propose to build, I earnestly hope that the undertaking, in which I take a deep personal interest, may effectually contribute to the relief of human suffering and the health of those whose career of honourable labour has been interrupted by accident or by sickness." "A deep personal interest" is professed by the Queen in the building of a new hospital, and she sees already, by anticipation, the wards occupied by men whose career in life is that of "honourable labour," and whose business has been interrupted "by accident or by sickness"; she also wishes that the new hospital may "effectually contribute" to the relief of suffering and to the restoration of health.

Fifty years' experience has taught us to believe in the woman as well as in the Queen. When she says that she takes a deep personal interest in the new hospital at Derby, we who read take her words for truth; when also she speaks of the working man's career as one of "honourable labour," we take that, too, as the expression both of a conviction, and of a sympathetic sentiment. Whoever takes the trouble to understand and estimate the value of things as they are in the immediate present, and as they are happening round about him every day, sees some reason for pleasure and satisfaction in the Queen's work and words at Derby. It is not a very unusual thing that a new hospital should be built in England; nor is it very wonderful that the head of the State should make a journey to take part in the proceedings. Not wonderful: no. But it might have been otherwise. It has been otherwise, and very much otherwise in this country; and even to-day it is very much otherwise in some countries. We cannot imagine our first William, truly called "The Conqueror," laying the foundation stone of a hospital for the sick poor; and speaking of their unremitting toil, with which he personally was so unfamiliar, as "honourable labour." The great Tudor Henry, the eighth of that name, and his still greater daughter Elizabeth, would probably have felt it a strange occupation to have laid the foundation stone of a poor man's hospital, and would no doubt, have smiled inwardly, with something perhaps of a cynical relish, if they had found themselves speaking of the despised workman's toil as "honourable labour."

There is a mighty change since those stalwart times, and we may as well note it in regard to matters medical and charitable, as in regard to the affairs of religion and high politics. Men and women feared "The Conqueror," who "made a desolation and called it peace"; they feared the great Cardinal's master, too: and even Elizabeth, whom they admired so much, they admired with trembling. We can contrast our Queen, a Tudor herself, with those other great English monarchs; and the contrast is one of the most significant demonstrations that could be made of the happiness of the new times as compared with the old. Men, as well as women, and men of the soundest brains and of the widest vision love Queen Victoria, and venerate her name. Long may she reign.

The hospital at Derby and other such works may appropriately be compared with the desolation which the Conqueror made, when some of his Saxon subjects ventured to question his right to reign. For sixty miles north of the Midlands, from Derby almost to Newcastle he laid waste the country, burnt the homesteads with the reaped and standing crops, and slew with the sword or reduced to poverty the greater part of the inhabitants of that North Midland country. There may be people who question Queen Victoria's right to reign; there are people who question anybody's right to reign, unless, perhaps, they have a very deep and hidden reservation in favour of themselves. But the Queen treats this constructive conspiracy in a different fashion from William's method; she lays the foundation of a hospital; she speaks words of honourable sympathy to her toiling fellow men; and she passes on her peaceful way to the quiet and unarmed safety of her northern home.

The hospital itself that is to be built has begun its career in the sunshine. The town and the county have been loyal and generous to the cause of medical charity in an extraordinary degree. The pace at which the movement has been carried on has been wonderful. Less than two years ago a new hospital was hardly contemplated. Circumstances, however, forced the subject forward; the managers considered several excellent schemes and chose the best. The public, from the highest to the lowest, welcomed their choice with enthusiasm. It was resolved to spend the large sum of seventy thousand pounds; and already, within a single year, nearly fifty thousand pounds have been promised or paid. When the work of Derby is compared with that of North London, which contains a population as great or greater than that of Derby, the grand generosity and business capacity of the former are seen in effective contrast. North London has been trying to build a new hospital for twenty years at a cost of forty thousand pounds. It has succeeded in building only half its hospital a cost of £20,000. Derby resolves to build a hospital at a cost of seventy thousand pounds; and in less than two years succeeds in raising more than forty thousand towards that grand total. In other words, the generosity and business capacity of Derby, as illustrated by its works of charity, are exactly twenty times greater than the generosity and business capacity of North London.

But what of Oxford, the county of wealthy residents; the city of colleges and towers; the mother of universities? Seven years ago, her great and proud spirit rose to the majestic height of resolving to spend £10,000 on her hospital. She resolved but never accomplished. The work to-day is still undone—unfinished, and not even begun. On the contrary, the Oxford managers are in a condition of peevish irritability, perhaps even of obstinate sulkiness because they have been reminded of their poor promises, and their still poorer fulfilments. But this cannot be permitted to continue. Oxford must have her duty persistently placed before her. Like Derby she must have her generous enthusiasm roused; she must resolve upon a hospital worthy of herself, and worthy of the Queen's help as the prime builder. If Oxford resolves to build herself a new hospital, worthy of the ci y and the county, there can be no doubt that Her Majesty the Queen, as she did for Derby, will put the first stone in its place, and the building will thus become, perhaps, the principal Oxford memorial of the Victorian period. But the Oxford managers must remember that the Queen is no longer young; and therefore, if the re-building of the hospital is to be done at all, it were well that it be done quickly.

